# The Differential Effects of Chronic Alcohol and Cigarette Smoke Exposures on Cognitive-Behavioral Dysfunction in Long Evans Rats

**DOI:** 10.4236/jbbs.2022.129024

**Published:** 2022-09-20

**Authors:** Emine B. Yalcin, Büşra Nur Delikkaya, William Pelit, Ming Tong, Suzanne M. De La Monte, Sharon Rounds

**Affiliations:** 1 Division of Research, Providence VA Medical Center, Providence, RI, USA; 2 Liver Research Center, Division of Gastroenterology and Department of Medicine, Rhode Island Hospital and the Alpert Medical School of Brown University, Providence, RI, USA; 3 Chemical Biology and English, Brown University, Providence, RI, USA; 4 Departments of Medicine, Neurology, and Pathology and Laboratory Medicine, Rhode Island Hospital, Women & Infants Hospital of Rhode Island, Alpert Medical School of Brown University, Providence VA Medical Center, Providence, RI, USA; 5 Departments of Medicine and Pathology and Laboratory Medicine, Warren Alpert Medical School of Brown University, Vascular Research Laboratory, Providence VA Medical Center, Providence, RI, USA

**Keywords:** Alcohol, Cigarette Smoke, Spatial Learning, Recognition Memory, Anxiety, White Matter, Rat

## Abstract

**Background and Objective:**

Chronic heavy alcohol consumption and daily cigarette smoking are the most prevalent substance use problems in the U.S., including Veterans. Excessive alcohol use causes neurocognitive and behavioral deficits that can be linked to neurodegeneration. Similarly, preclinical and clinical data suggest that smoking also leads to brain atrophy. This study examines the differential and additive effects of alcohol and cigarette smoke (CS) exposures on cognitive-behavioral function.

**Methods:**

A 4-way experimental model of chronic alcohol and CS exposures was generated using 4-week-old male and female Long Evans rats that were pair-fed with Lieber-deCarli isocaloric liquid diets containing 0% or 24% ethanol for 9 weeks. Half of the rats in the control and ethanol groups were exposed to CS for 4 hours/day and 4 days/week for 9 weeks. All rats were subjected to Morris Water Maze, Open Field, and Novel Object Recognition testing in the last experimental week.

**Results:**

Chronic alcohol exposure impaired spatial learning as shown by significantly increased latency to locate the platform, and it caused anxiety-like behavior marked by the significantly reduced percentage of entries to the center of the arena. Chronic CS exposure impaired recognition memory as suggested by significantly less time spent at the novel object. Combined exposures to alcohol and CS did not show any significant additive or interactive effect on cognitive-behavioral function.

**Conclusion:**

Chronic alcohol exposure was the main driver of spatial learning, while the effect of secondhand CS exposure was not robust. Future studies need to mimic direct CS exposure effects in humans.

## 1. Introduction

The most prevalent types of substance use problems among male and female veterans include chronic heavy alcohol consumption and daily cigarette smoking [1]. Relative to civilians, veterans have higher prevalence rates of lifetime alcohol and tobacco use disorders and lower physical and cognitive-behavioral functions [2] [3]. Although there are several behavioral and pharmacological treatments to help reduce substance use among veterans, only a minority receive treatment and few report unmet need for treatment of substance use disorders [1] [4]. In addition, neurobiological changes in people detoxifying from alcohol or tobacco can increase the risk of relapse even after protracted abstinence, a hallmark of addiction [5]. Therefore, mechanism-driven preventive and harm-reduction therapeutic measures are needed to enhance the quality of life for Veterans and their families.

The damaging effects of chronic heavy alcohol consumption on brain structure and function have been well studied over recent decades. Considerable research in humans has demonstrated that chronic alcohol consumption can cause severe adverse effects on the brain leading to deficits in cognitive function including decreased learning and memory [6] [7], divided attention [8], decision making [9], and problem solving [10]. Postmortem and neuroimaging studies have correlated these functional deficits with structural abnormalities and reported that white matter in the corpus callosum, prefrontal area, temporal lobe, and cerebellum was disproportionately affected by alcohol [11] [12]. Furthermore, more recent experimental studies linked chronic plus binge alcohol-induced cognitive decline with white matter atrophy and myelin degeneration [13] [14]. Growing evidence suggests that alcohol can also disrupt brain development during the adolescent period, and there may be potential gender differences in the impact of alcohol on cognitive function [15] [16] [17]. Several studies indicated that females are more susceptible to alcohol-induced brain damage than males; however, there is not enough evidence to state the gender effect conclusively [15] [16] [17] [18].

Cumulative evidence suggests that chronic heavy smoking has neurotoxic effects on the brain and is associated with impaired cognition and increased risk for dementia in adults [19] [20]. In addition, the adolescent brain is particularly sensitive to adverse effects of smoking on cognitive performance [21]. Although preclinical models and human studies demonstrated that nicotine has cognition enhancing effects such as improved motor functions, attention, and memory [22], there are many harmful components in both mainstream (*i.e.*, smoke inhaled by active smokers) and side stream (*i.e.*, smoke inhaled by passive smokers) tobacco smoke that can damage every organ, tissue, or cell type. Cigarette smoke contains over 7000 chemical compounds such as highly reactive, volatile aldehydes, tar, polycyclic aromatic hydrocarbons, arsenic, benzene, carbon monoxide, heavy metals, and tobacco-derived nitrosamines [23]. Additionally, every puff of cigarette smoke contains nitrogen-, carbon-, and oxygen-centered radicals and produces esters and peroxyesters of nitrous and nitric acid in the gas phase that disrupt redox signaling and increase oxidative stress globally [24]. Previous studies have shown that chronic exposures to the tobacco-specific nitrosamine NNK cause white matter atrophy and cognitive impairment at a submutagenic dose [14] [25]. Additional studies are necessary to address the long-term neurocognitive and neurodegenerative effects of other chemical toxins involved in cigarette smoke.

Excessive alcohol and tobacco use are closely linked behaviors, nearly 80% of heavy alcohol users smoke and vice versa. Due to the frequent occurrence of concurrent dependence, interactions between alcohol and tobacco have received growing attention from basic and clinical researchers over the past decade. Previous studies mainly focused on the consequences of heavy drinking on brain structure and function, and much less is known about the neurocognitive deficits of chronic smoking. Furthermore, a number of studies suggest that gender might play a critical role in alcohol and cigarette smoke mediated neurotoxicity, yet limited studies incorporated both sexes in the experimental design [26] [27] [28] [29]. This study will investigate the individual and additive/interactive effects of chronic cigarette smoke and alcohol exposures on cognitive-behavioral functions in male and female adolescent Long Evans rats.

## 2. Methods

### 1) Animals:

3-week-old male and female Long Evans rats were obtained from Charles River Laboratories. Rats were group-housed in cages of two in a pathogen-free animal facility with an automated 12-hour light/dark cycle with free access to food. All procedures were carried out in compliance with the National Institutes of Health (NIH) Guide for the Care and Use of Laboratory Animals and were approved by the Institutional Animal Care and Use Committees (IA-CUCs) at the Providence VA Medical Center and Rhode Island Hospital.

### 2) *In Vivo* Experimental Model:

We generated a 4-way experimental model of chronic alcohol and CS exposures using 4-week-old male and female Long Evans rats (n = 8/group). Rats were pair-fed with Lieber-deCarli isocaloric liquid diets containing 0% or 24% ethanol for 9 weeks. Ethanol-fed rats were acclimated to the alcohol diet by gradually increasing the dose over 4 days. Half of the rats in the control and ethanol groups were exposed to CS using research grade Kentucky 1R6F cigarettes (Tobacco Research Institute, University of Kentucky, Lexington, KY) and an industry standard Teague Enterprises TE-10 Smoking Machine equipped with three exposure chambers (Woodland, CA). CS-exposed rats were rotated through one of three exposure chambers during each smoking session. The other half, air-exposed rats, were treated identically but without cigarettes. The smoking chamber atmosphere was monitored for total suspended particles (TSP) at a concentration of 150 mg/m^3^ by burning three cigarettes simultaneously. The smoke was a mixture of sidestream (89%) and mainstream (11%) smoke. Three cigarettes were puffed simultaneously for 2 seconds every minute for 9 minutes. The cigarettes were burned for 4 hours/day and 4 days/week for 9 weeks. A subgroup of CS- and air-exposed rats was fed with chow instead of the liquid diet.

### 3) Model Characterization:

Food intake and body weight were monitored daily to ensure weight gain throughout the experiment. During the last week of the experiment, rats were subjected to neurobehavioral tests including Morris Water Maze, Open Field, and Novel Object Recognition tests between 9 am and 3 pm with a minimum of a 24-hour interval between tasks. All rats were alert and active and showed no signs of intoxication during neurobehavioral testing. Following behavioral analysis, rats were euthanized by exsanguination via cardiac puncture under deep terminal isoflurane anesthesia. Blood alcohol concentrations were measured with a commercially available colorimetric assay kit (BioVision, California, USA). Serum cotinine levels were measured by a solid-phase competitive ELISA kit (OriGene, Rockville, MD). Blood glucose levels were measured by One Touch Ultra glucometer (LifeScan Inc, Milpitas, CA).

### 4) Morris Water Maze (MWM):

The MWM test was used to evaluate spatial learning. In brief, each rat was placed individually in a 6-foot diameter, white, polyethylene, seamless water maze (SD Instruments, San Diego, CA) filled with water colored opaque using non-toxic tempera paint (Dick Blick Art Materials, Galesburg, IL). The rats were allowed to swim and search for the platform for a maximum of 120 seconds. If the rats failed to find the platform within this time limit, they were guided toward the platform and allowed to sit on it for 15 seconds. The latency to find and land on the platform, path length, and speed were measured with EthoVision XT v16 software (Noldus Information Technology, Leesburg, VA). Each rat underwent three trials daily for four consecutive days. On the first day, rats were trained to find the visible platform, and on the following three days, they were tested to find the hidden platform submerged under opaque water. The start positions of rats were kept the same on the first two days, and they were randomized for each trial on the last two days. The area under the curve of latency, path length, and speed were calculated for the three daily trials for inter-group comparison.

### 5) Open Field (OF):

The OF test was used to evaluate the general locomotor activity, anxiety, and exploration behavior in rats. In brief, a white square (90 cm × 90 cm) acrylic box was used with a 4 × 4 grid of 22.5 cm squares covering the floor of the maze. Each rat was placed individually in the maze at the exact location facing the wall and allowed to explore the arena for 5 minutes while being recorded by an overhead camera. The footage was analyzed for the percentage of time rats spent in the center, percentage of entries to the center, latency to the center, and total distance traveled with EthoVision XT v16 software. The maze was wiped with 70% ethanol prior to use and before each trial to remove any scent clues, feces, and urine left by the previous subject rat. The room was illuminated by indirect white dim light provided with 8 bulbs.

### 6) Novel Object Recognition (NOR):

The NOR test was used to assess cognition, particularly recognition memory in rodents. The test includes 3 phases: habituation, training, and testing. For the habituation phase, each rat was introduced to the empty arena to explore freely for 5 minutes, which had been performed during the OF test. On the next day, training was conducted by placing each rat in the same arena that contained two identical objects to explore for 5 minutes. After 24 hours, long-term memory testing was conducted by returning each rat to the arena that contained one of the original objects and a new object to explore for 5 minutes. The original and new objects were built from LEGO^®^ pieces of similar size and texture but different composition of color and shapes. The arena and objects were cleaned with 70% ethanol after each trial. The footage was analyzed for the percentage of time rats spent in the objects and at the objects (within 2 cm distance) with EthoVision XT v16 software. The results were analyzed by calculating the recognition index (RI), *i.e.*, the time rats spent investigating the novel object relative to the total object investigation time.

### 7) Data Analysis:

The graphed data correspond to mean ± standard deviations. Intergroup comparisons were made first by three-way analysis of variance (ANOVA) with Tukey or linear trend post hoc comparisons using Graph Pad Prism version 9 (GraphPad Software, San Diego, CA) to determine alcohol, CS, and gender effects. When there was no gender effect, the data from each sex was combined and re-analyzed by one- or two-way ANOVA with Tukey or linear trend post hoc tests. F ratios and P values are presented. Significant differences calculated by Tukey post hoc tests (P < 0.05) and trends (0.05 < P < 0.10) are demonstrated on the graphs.

## 3. Results

### 1) Chronic Alcohol and/or Cigarette Smoke Exposure Model Characteristics:

**Figure 1.**
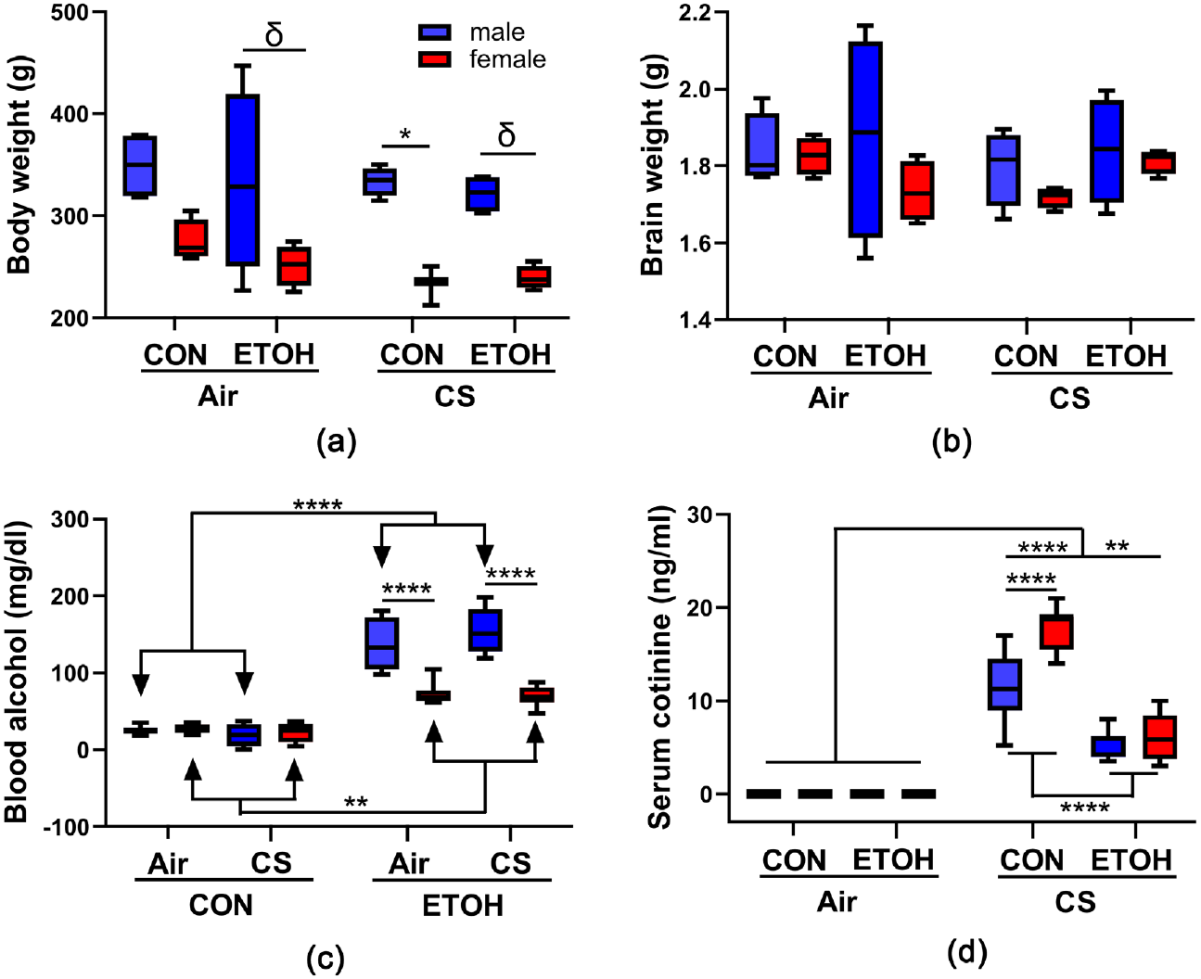
Effects of chronic ethanol and cigarette smoke exposures on (a) body weight, (b) brain weight, (c) blood alcohol, and (d) serum cotinine: A 4-way experimental model generated with male and female Long Evans rats was grouped as follows: CON: control diet + air exposure; ETOH: ethanol diet + air exposure; CS: control diet + CS exposure; and ETOH + CS: ethanol diet + CS exposure (n = 8/group). The box and whisker plots depict the mean and 95% confidence intervals of the parameters. Inter-group comparisons were made by three-way ANOVA with the Tukey post hoc tests (*P < 0.05; **P < 0.01; ***P < 0.001; ****P < 0.0001; *δ* 0.05 < P < 0.10).

All rats consumed their diet daily and continuously gained weight throughout the study (Supplementary Figure S1(a)-(c)). Three-way ANOVA revealed that gender had a significant effect on body weight measured at sacrifice, and post hoc Tukey tests demonstrated that males had significantly higher mean body weights than females (Table 1, Figure 1(a)). Brain weights (Figure 1(b)) and blood glucose levels (data not shown) did not differ significantly among the groups. Blood alcohol concentrations were significantly higher in ethanol-fed male (P < 0.0001) and female (P = 0.003) rats relative to controls, and males had significantly higher mean alcohol levels than females (P < 0.0001) (Figure 1(c)). Serum cotinine levels were significantly higher in CS (P < 0.0001) and CS+ ethanol exposed (P = 0.002) rats relative to air exposed rats (Figure 1(d)). The total suspended particulate (TSP) concentrations in three chambers ranged from 68.8 - 189.6 mg/m^3^; however, the mean of total TSP levels exposed by each group of rats was comparable (Supplementary Figure S2).

A subgroup of control and CS-exposed rats was fed with chow since diet could serve as a potential confounding factor. All chow-fed rats gained weight daily throughout the exposures (Supplementary Figure S3(a)). Body weights measured at sacrifice were significantly higher in chow-fed rats relative to liquid-fed counterparts (P = 0.0002) (Supplementary Figure S3(b)). Diet had no significant effect on brain weights and blood glucose levels (Supplementary Figure S3(c) and Figure S3(d)). Serum cotinine levels were significantly higher in chow-fed male rats relative to liquid-fed males (P < 0.0001) after 9 weeks of chronic CS exposures, while no difference was observed in females (Supplementary Figure S3(e)).

### 2) Neurobehavioral Analysis:

#### Morris Water Maze:

**Figure 2.**
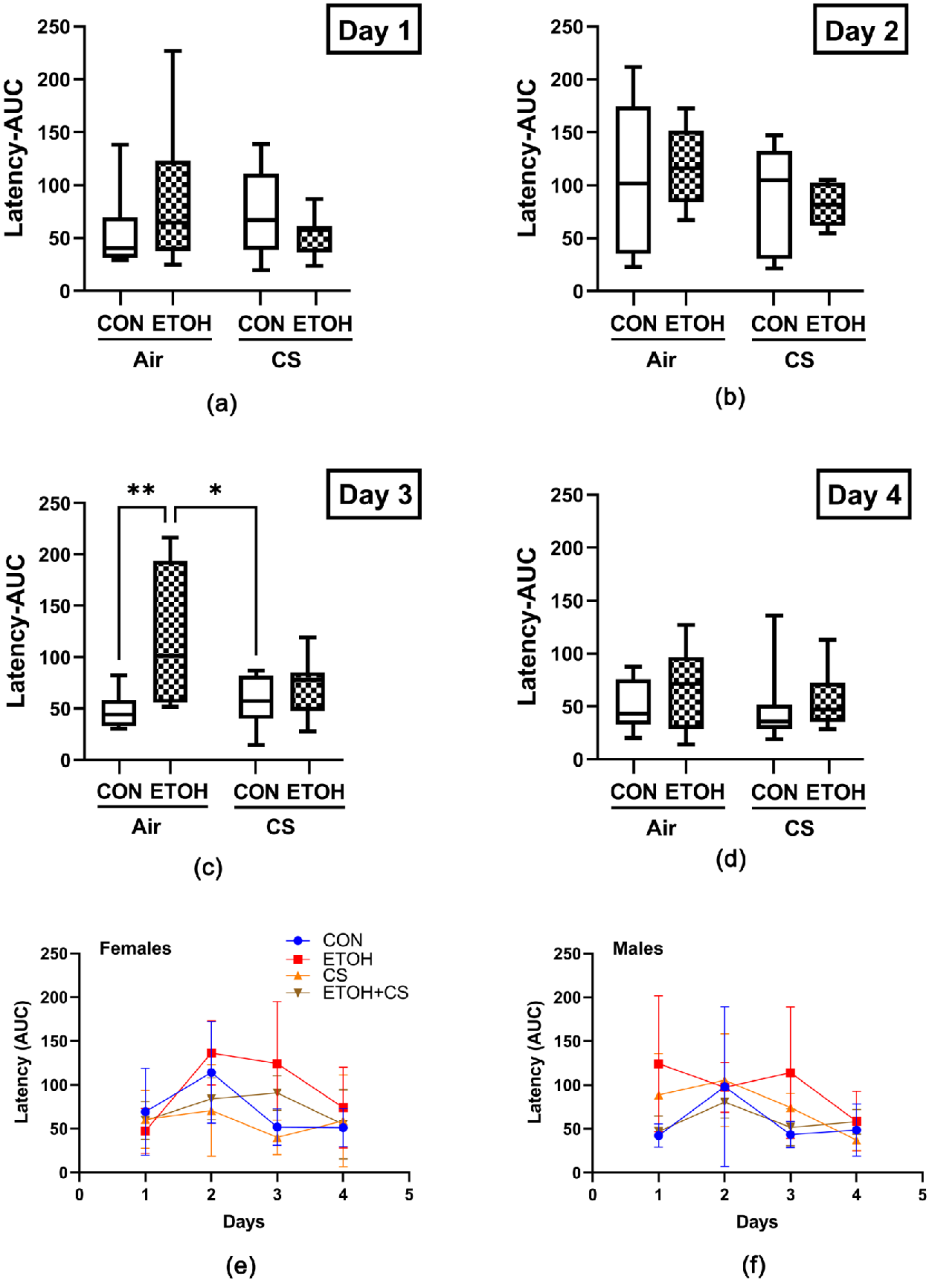
Morris Water Maze (MWM): A 4-way experimental model generated with male and female Long Evans rats was grouped as follows: CON: control diet + air exposure; ETOH: ethanol diet + air exposure; CS: control diet + CS exposure; and ETOH + CS: ethanol diet + CS exposure. MWM test was performed over 4 days (Monday-Thursday) with 3 trials per day after 8 weeks of ethanol ± CS exposures. Data were analyzed by calculating the area under the curve (AUC) for latencies to locate the platform on (a) Day 1; (b) Day 2; (c) Day 3; and (d) Day 4. The box and whisker plots depict the mean and 95% confidence intervals of the parameters. Inter-group comparisons were made by two-way ANOVA with the Tukey post hoc tests (*P < 0.05; **P < 0.01).

The MWM test demonstrated alcohol and/or CS mediated impairments in spatial learning by measuring the latency and path length to locate a submerged escape platform. All rats exhibited the most prolonged mean latency on day 2, the first day of testing with the platform submerged below the water surface (Figure 2(b)). On trial days 3 and 4, the latencies were improved for all groups although they were still prolonged in the ethanol relative to control (Figure 2(c) and Figure 2(d)). Post hoc linear trend analysis demonstrated that progressive improvement of latencies was statistically significant in control female rats and CS-exposed male rats over time (from day 2 to day 4), whereas ethanol and ethanol + CS groups failed to reach statistical significance (Table 2 and Figure 2(e) and Figure 2(f)). Inter-group comparisons revealed significant ethanol effects and ethanol × CS interactions on Day 3 as demonstrated by two-way ANOVA (Table 3). Post hoc Tukey tests revealed significantly longer mean latency in ethanol-exposed rats relative to control (P = 0.006) and CS-exposed rats (P = 0.02) (Figure 2(c)). In conjunction with latency, the path length was longer in the ethanol group as compared to the control and CS groups on Day 3, whereas the velocities of rats remained the same among the groups (data not shown).

#### Open Field (OF):

The OF test demonstrates individual and combined effects of alcohol and CS on rat exploration behavior, anxiety, and gross locomotor activity by measuring the latency to the center of the arena, time spent in the center, number of entries to the center, and the distance traveled by rats. Chronic alcohol and/or CS exposures did not have a significant effect on the anxiety measures potentially due to rats’ increased body size (210 - 356 g) after 8 weeks of exposures which requires a larger arena (data not shown). However, we were able to detect alcohol-mediated effects on anxiety with younger rats (109 - 161 g) even after a short-term (2 weeks) exposure. Representative heatmaps generated by EthoVision software demonstrated that control and CS-exposed rats traveled freely in the arena including the center zone, whereas ethanol and ethanol + CS-exposed rats remained mainly in the outer zone and crossed the center of the arena only a few times (Supplementary Figure S4(e)). Two-way ANOVA revealed that two weeks of chronic ethanol consumption had significant effects marked by lower mean percentages of time spent in the center of the arena, lower mean percentage of entries to the center, and increased latency to the center (Supplementary Table S1). Tukey post hoc tests demonstrated that ethanol- and ethanol + CS-exposed rats had a significantly lower percentage of entries to the center relative to CS-exposed rats (Supplementary Figure S4(b)). The movement distance increased significantly in alcohol, CS, and dual exposed rats relative to the control group (Supplementary Figure S4(d) and Supplementary Table S1). In addition, the chow versus liquid diet comparison resulted in no significant change in any parameters of the OF test (data not shown).

#### Novel Object Recognition (NOR):

**Figure 3.**
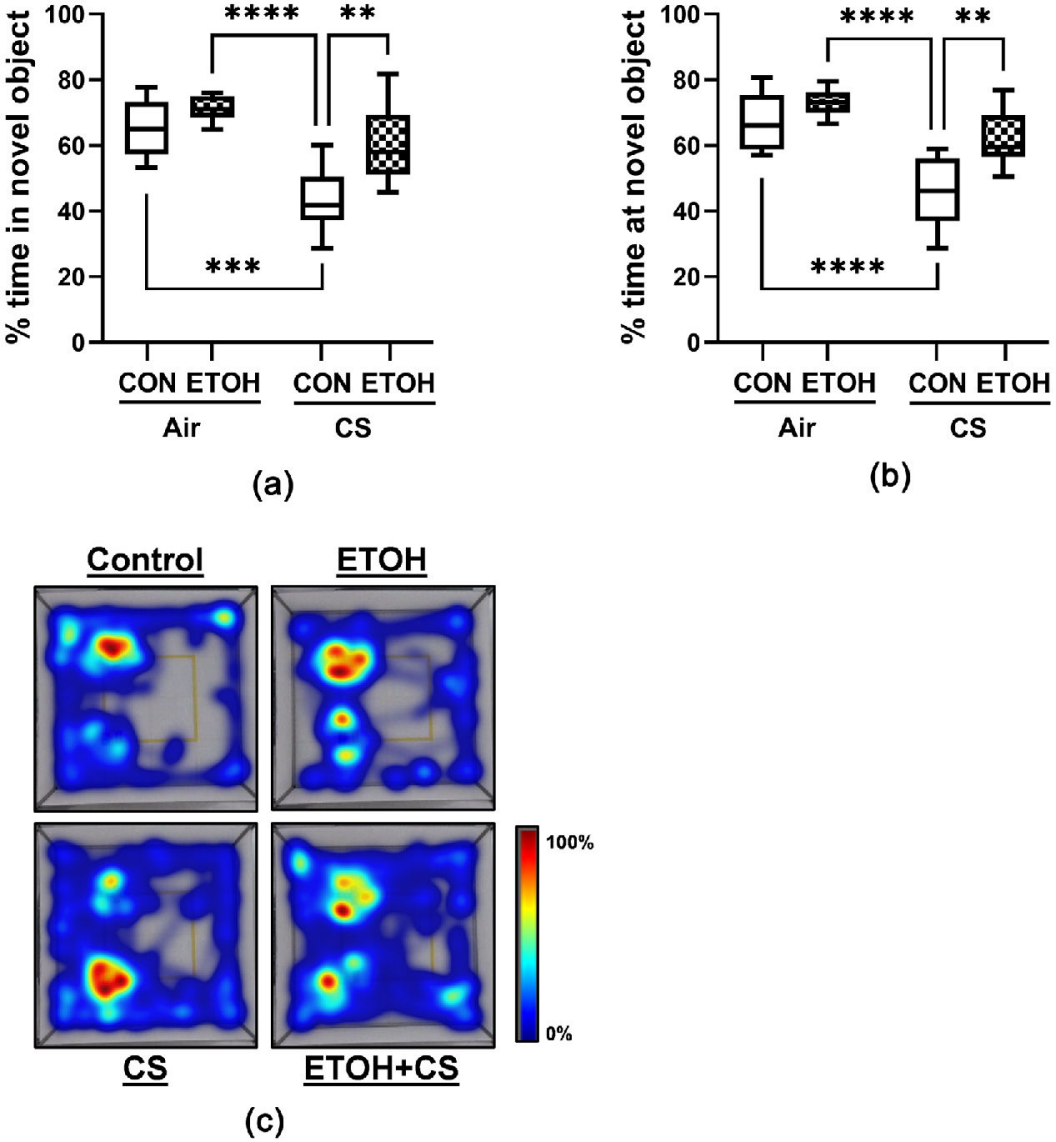
Novel Object Recognition (NOR): A 4-way experimental model generated with male and female Long Evans rats was grouped as follows: CON: control diet + air exposure; ETOH: ethanol diet + air exposure; CS: control diet + CS exposure; and ETOH + CS: ethanol diet + CS exposure. NOR test was performed after 8 weeks of exposure and data were analyzed by EthoVision XT v16 software with respect to the (a) time spent in the novel object and (b) time spent at (surrounding) the novel object. (c) EthoVision generated heatmaps depict location of each rat in the arena during analysis. The box and whisker plots depict the mean and 95% confidence intervals of the parameters. Inter-group comparisons were made by two-way ANOVA with the Tukey post hoc tests (**P < 0.01; ***P < 0.001; ****P < 0.0001).

The NOR test evaluated alcohol-, CS-, and alcohol + CS-mediated alterations in recognition memory by measuring the time spent at the novel object normalized to the total investigation time spent at novel and familiar objects. During the training phase, all rats interacted with both objects and exhibited no signs of preference for any objects. However, during the testing phase, CS-exposed rats exhibited a shorter interaction with the novel object, as shown in heatmaps (Figure 3(c)). Two-way ANOVA revealed significant ethanol and CS effects on % time spent in or at the novel object, and a trend effect for ethanol x CS interaction (Table 4). Post hoc Tukey tests demonstrated that CS-exposed rats spent significantly less time at or in the novel object relative to control (P = 0.0002), ethanol- (P < 0.0001), and dual-exposed (P = 0.004) rats (Figure 3(c) and Figure 3(d)). The adverse effects of smoking on recognition memory were not observed in chow-fed rats (data not shown).

## 4. Discussion

This study aimed to assess individual and combined effects of chronic alcohol and cigarette smoke exposures on cognitive-behavioral functions. Previous studies have shown that individual exposures to chronic alcohol and cigarette smoke produce white matter degeneration, myelin loss, and cognitive impairment in rats [13] [14] [25] [30] resembling human alcohol and tobacco use disorders [11] [20] [31] [32] [33] [34]. Although it is well established that alcohol consumption and tobacco use are highly correlated (80% to 95% of heavy alcohol users also smoke), previous experimental and clinical studies mainly addressed only one substance. Effective treatment entails a better understanding of how alcohol and tobacco interact; therefore, we paired smoking with alcohol exposure in the same rat strain to assess responses to different exposures in relation to the severity of cognitive-behavioral dysfunction. Previous reports dating back nearly two decades demonstrate that gender plays a significant role in alcohol mediated brain injury, suggesting that females are more vulnerable to neurotoxic effects than males identified by different neuroimaging modalities [26] [27] [28]. However, research is limited due to low female enrollment and underpowered analysis to account for gender differences [29]. Our findings demonstrated that female rats were vulnerable to alcohol-induced impairment in spatial learning. In addition, blood alcohol concentrations were higher in male rats, whereas serum cotinine levels were higher in females suggesting a gender difference in pharmacology and metabolism of these toxins. Interestingly, we detected a significant ethanol effect on serum cotinine levels, which were significantly lower in ethanol + CS-exposed rats relative to the ethanol group suggesting a potential cofactor effect of alcohol on nicotine metabolism. Indeed, individuals with alcohol use disorders have a much higher rate of nicotine metabolism and greater CYP2A6 enzyme activity than non-alcoholic smokers [35]. Consequently, alcohol-dependent individuals are distinguished as heavier smokers, report higher nicotine dependence, and have lower smoking cessation rates [36] [37].

Chronic alcohol and cigarette smoke exposures produced distinct effects on cognitive-behavioral dysfunction in Long Evans rats. Alcohol impaired spatial learning as determined by Morris Water Maze, one of the “gold standards” of behavioral neuroscience. There is ample evidence that the hippocampus is critically involved in learning and memory [38]. Furthermore, studies have increasingly recognized the importance of the white matter in cognition [39] [40]. Early studies showed that alcohol’s neurotoxic effects on the white matter include demyelination, dysmyelination, and axonal degeneration [41]. Chronic CS exposure produced similar pathologies in the white matter of an adult mouse model; therefore, we expected to observe impairment in cognitive function following chronic CS exposures. However, CS-exposed rats performed similarly to controls by Morris Water Maze testing. This might be due to the lower dose/durations of CS exposures (4 hours/day, 4 days/week) used in our rat model relative to the previous study (6 hours/day, 5 days/week) [30]. Higher doses of CS or an alternative way of exposure may be necessary to assess smoking-induced deficits in spatial learning. Alternatively, the progressive improvement of latencies in control and CS-exposed male rats could be explained by the learning and memory enhancing effects of nicotine exposure in adolescent and aged rats [42].

Chronic alcohol exposure also caused anxiety-like behavior in rats as determined by their preference to remain in the periphery of the arena near the walls (thigmotaxis), which is considered a relatively safer area [43]. These findings are consistent with previous observations, which demonstrated relationship between ethanol intake and anxiety in animal studies [44] [45] [46] and humans [47]. In addition, the locomotor activity was significantly increased in ethanol- and CS-exposed rats relative to controls. Previous studies linked the enhancement of motor activity with ethanol-mediated reduction of the aversive effect of the novel environment, an innate response to reduce anxiety (anxiolytic effect) [48]. In contrast to the 2-week model, there were no measures of anxiety in the 8-week alcohol-exposed rats. The failure in evaluating the anxiety could be due to increased size of rats after 8 weeks of exposure and the necessity of using a larger arena (122 cm^2^) as in previous studies [49].

The cumulative research suggests that heavy smoking is associated with impairments in learning and memory, cognitive flexibility, working memory, and executive functions in young, middle-aged, and older adult populations [21] [33] [50] [51] [52]. In this study, we demonstrated that chronic CS exposure resulted in impairments in recognition memory in rats, as revealed by the novel object recognition test. Neuroimaging studies correlated smoking-mediated functional deficits with cerebral white matter atrophy [20] [32]. Furthermore, white matter degeneration has been shown in an adult mouse model following a similar duration of cigarette smoke exposure [30]. Future studies will investigate white matter histopathology in Long Evans rat brains to understand the mechanisms underlying smoking-mediated impairment in recognition memory.

Despite the high comorbid instances of alcohol and tobacco use disorder, only a few studies incorporated both toxins in the experimental design. A major advantage of this study lies in the approach, which is designed to pair chronic CS exposures with alcohol in the same strain to generate a more clinically relevant model. The inclusion of the chow diet as a confounding factor is another strength of this research. One limitation of this study could be associated with smoke exposure as rats were exposed mainly (89%) to sidestream smoke which mimics secondhand smoking. In humans, blood cotinine levels are always >10 ng/mL in active smokers and they can reach to 500 ng/mL, while the levels are <10 ng/mL in secondhand smokers and <1 ng/mL in non-smokers [53]. In our experimental model, serum cotinine levels in CS exposed rats were ranged between 5 - 25 ng/mL, confirming that the model largely mimics secondhand smoke exposure. Although CS exposure doesn’t imitate active smokers, the secondhand smoke exposure is relevant as epidemiological studies estimated that 20.8% of non-smoking U.S. adults aged > 18 were exposed to secondhand smoke as measured by cotinine in the blood [54], and secondhand smoking has been associated with increased risk of cognitive impairment and dementia [55].

## 5. Conclusion

In conclusion, this study demonstrated chronic alcohol and/or secondhand cigarette smoke exposures on cognitive-behavioral functions including learning, recognition memory, anxiety, and exploration in adolescent Long Evans rats. The results show that alcohol was the main driver of impairment in spatial learning while cigarette smoke alone or in combination with alcohol did not have a robust effect. Future studies should improve CS exposure to produce a more clinically relevant model that resembles human smokers.

## Supplementary Material

1

## Figures and Tables

**Table 1.** Ethanol, CS, gender, and interactive effects on the experimental model characteristics.

	Ethanol Effect		CS Effect		Gender Effect		Ethanol × CS × Gender Interaction	
	F Ratio	P value	F Ratio	P value	F Ratio	P value	F Ratio	P value
Body weight	0.716	ns	2.176	ns	**38.46**	**<0.0001**	0.224	ns
Brain weight	0.228	ns	0.363	ns	2.266	ns	1.134	ns
Blood glucose	1.248	ns	2.019	ns	2.694	ns	<0.0001	ns
Blood alcohol	**235.6**	**<0.0001**	0.035	ns	**42.16**	**<0.0001**	1.217	ns
Serum cotinine	**43.95**	**<0.0001**	**212.9**	**<0.0001**	**7.701**	**0.008**	3.766	ns

Three-Way ANOVA results from comparing mean levels of body weight, brain weight, blood glucose, blood alcohol concentrations, and serum cotinine levels in control, ethanol-, CS-, and ethanol + CS-exposed male and female Long Evans rats. Significant differences are highlighted with bold font. Corresponding data with Tukey post-hoc significance test outcomes are graphed in Figure 1.

**Table 2.** Morris water maze: Linear trend analysis.

Gender	Groups	F Ratio	P value	Slope	R square
Females	Control	**5.54**	**0.04**	**−31.46**	**0.758**
	Ethanol	2.753	ns	−31.16	0.888
	Cigarette Smoke	0.146	ns	−5.969	0.148
	Ethanol + Cigarette Smoke	2.045	ns	−14.66	0.588
Males	Control	1.563	ns	−24.76	0.672
	Ethanol	1.193	ns	−19.4	0.466
	Cigarette Smoke	**8.821**	**0.02**	**−34.15**	**0.998**
	Ethanol + Cigarette Smoke	3.099	ns	−11.25	0.550

The latencies in arriving on the platform were measured and the area under curve results were used for intra-group comparisons (Figure 2(e) and Figure 2(f)). Linear trend analysis was used to test if the decreasing latencies of control, ethanol, cigarette smoke, and ethanol + cigarette smoke over 3 testing days were statistically significant in female (first 4 rows) and male (last 4 rows) rats. The F ratio, linear P value, trend slope, and calculated R square are indicated for each group. Significant differences are highlighted with bold font.

**Table 3.** Ethanol, CS, and interactive effects on spatial learning.

	Ethanol Effect		CS Effect		ETOH × CS Interaction	
	F Ratio	P value	F Ratio	P value	F Ratio	P value
Latency (AUC)-Day 1	0.074	ns	0.198	ns	2.677	ns
Latency (AUC)-Day 2	0.021	ns	2.303	ns	0.233	ns
Latency (AUC)-Day 3	**9.34**	**0.005**	1.87	ns	**4.22**	**0.049**
Latency (AUC)-Day 4	1.180	ns	0.257	ns	0.125	ns

Two-Way ANOVA results from comparing mean levels of the latencies (determined by the area under the curve analysis of three daily trials) to locate the platform. Significant differences are highlighted with bold font. Corresponding data with Tukey post-hoc significance test outcomes are graphed in Figures 2(a)-(d).

**Table 4.** Ethanol, CS, and interactive effects on novel object recognition.

	Ethanol Effect		CS Effect		ETOH × CS Interaction	
	F Ratio	P value	F Ratio	P value	F Ratio	P value
% time in novel object	**13.46**	**0.001**	**26.74**	**<0.0001**	2.899	0.1
% time at novel object	**13.42**	**0.001**	**30.96**	**<0.0001**	3.081	0.09

Two-Way ANOVA results from comparing mean levels of the percentage of time rats spent in the novel object and the percentage of time rats spent at (surrounding) the novel object. Significant differences are highlighted with bold font. Corresponding data with Tukey post-hoc significance test outcomes are graphed in Figure 3.
