# Supplementary material for: The Differential Effects of Chronic Alcohol and Cigarette Smoke Exposures on Cognitive-Behavioral Dysfunction in Long Evans Rats
Source: J Behav Brain Sci. Author manuscript; Available in PMC 2023 Feb 28. (PMC9974143; doi:10.4236/jbbs.2022.129024)
Supplement: 1 [file NIHMS1869473-supplement-1.pdf]

Supplementary

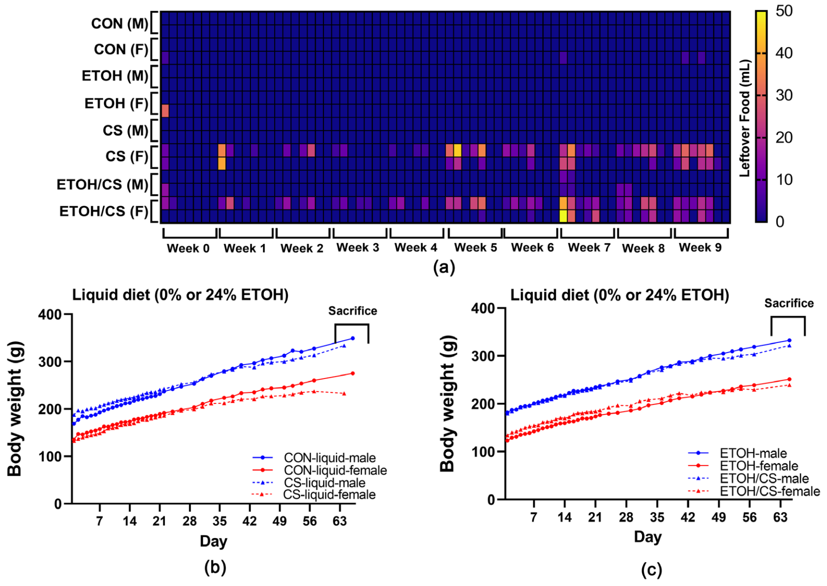

Figure S1. Effects of chronic alcohol and cigarette smoke exposures on food intake and body weight in male and female Long Evans rats. A 4-way 9-week model generated in male and female Long Evans rats was grouped as follows: CON: control diet + air exposure; ETOH: ethanol diet + air exposure; CS: control diet + CS exposure; and ETOH + CS: ethanol diet + CS exposure. (a) Food intake and ((b), (c)) daily body weights were measured to compare ethanol effects with or without CS exposure.

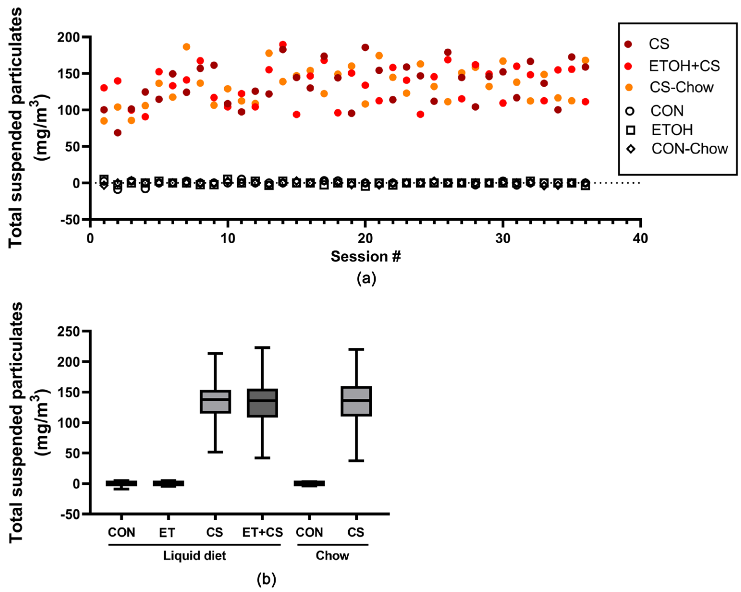

Figure S2. Total Suspended Particulate (TSP) Measurement: The smoking chamber atmosphere was monitored for TSP by sampling smoke on a filter paper for 5 minutes and measuring the exact volume of air passing through the filter paper with the dry test meter. The weight change of the filter paper is divided by the air volume to determine TSP (mg/m³). TSP was measured (a) at each smoking session and (b) the mean ± SD of TSP was calculated to compare the total smoke exposure of each group throughout the experiment.

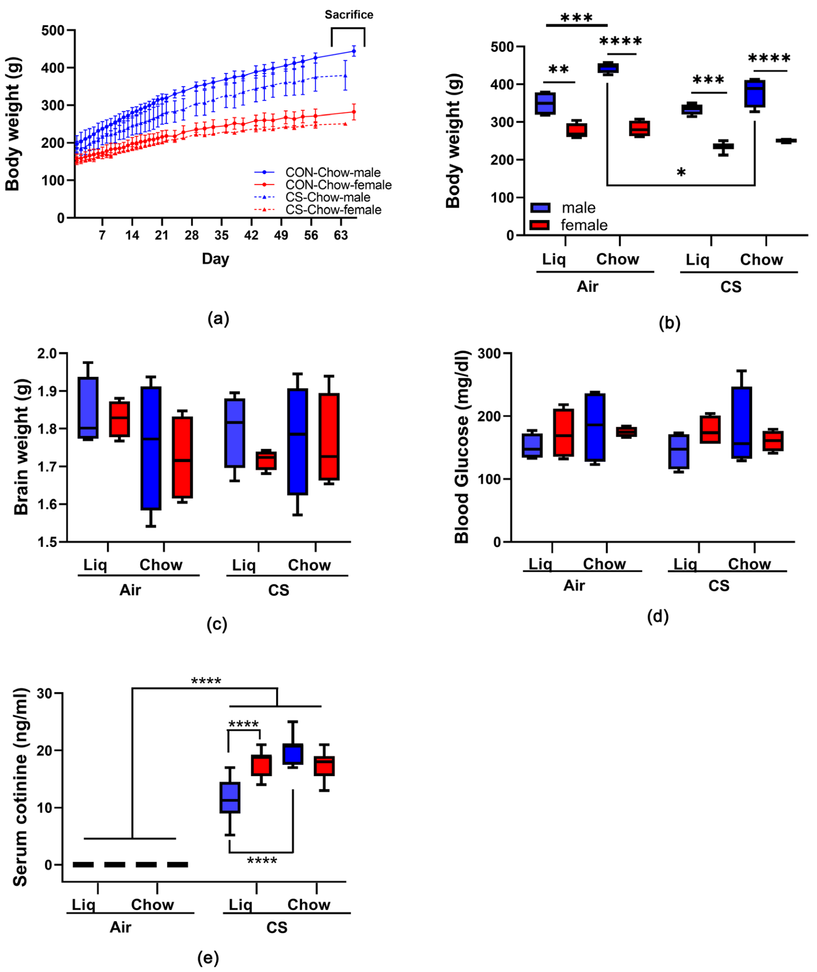

Figure S3. Effects of diet (chow versus liquid) and cigarette smoke exposures on (a) daily body weight, (b) body weight measured at sacrifice, (c) brain weight, (d) blood glucose, and (e) serum cotinine: A 4-way 9-week model generated in Long Evans rats was grouped as follows: Liq: control (liquid) diet + air exposure; Chow: chow diet + air exposure; Liq-CS: control (liquid) diet + CS exposure; Chow-CS: chow diet + CS exposure. The box and whisker plots depict the mean and 95% confidence intervals of the parameters. Inter-group comparisons were made by three-way ANOVA with the Tukey post hoc tests (*P < 0.05; **P < 0.01; ***P < 0.001; ****P < 0.0001).

Table S1. Ethanol, CS, and interactive effects on anxiety.

	Ethanol Effect		CS Effect		ETOH × CS Interaction	
	F Ratio	*P value*	*F Ratio*	*P value*	*F Ratio*	*P value*
% time in center	**7.126**	**0.01**	0.238	ns	0.873	ns
% entries to center	**14.14**	**0.0009**	0.244	ns	0.763	ns
Latency to center	**4.665**	**0.04**	0.731	ns	0.067	ns
Distance traveled	**20.09**	**0.0001**	**6.770**	**0.02**	3.263	ns

Two-Way ANOVA results from comparing mean levels of the percentage of time rats spent in the center of the open field, the percentage of entries into the center, the latency to the center (sec), the movement distance (cm) in the open field. Significant differences are highlighted with bold font. Corresponding data with Tukey post-hoc significance test outcomes are graphed in Figure S4.

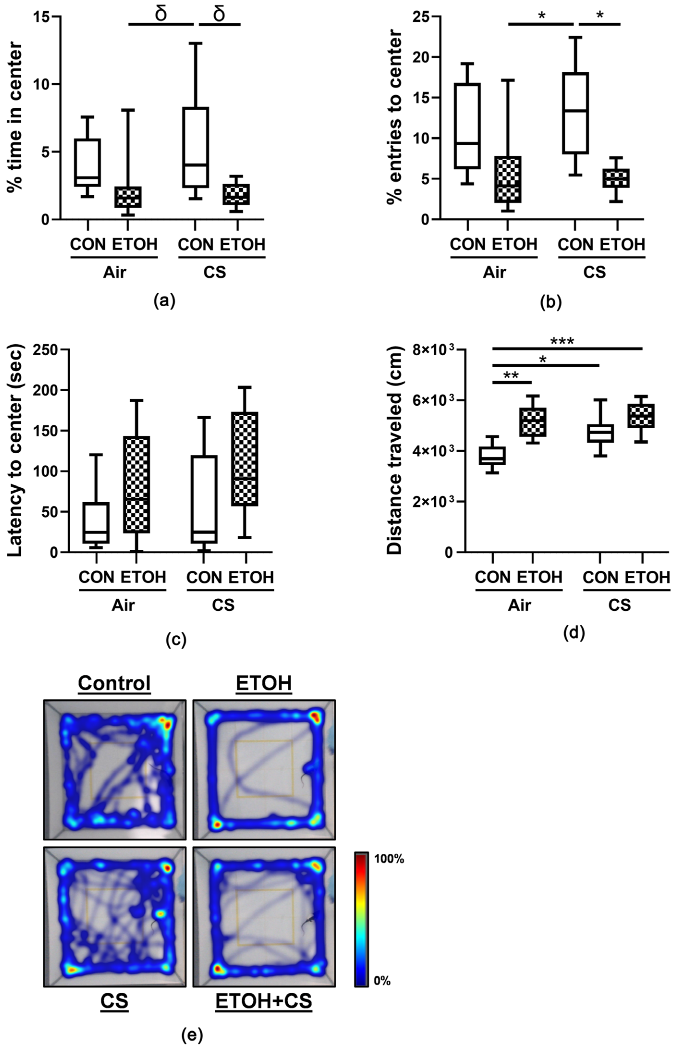

Figure S4. Open Field (OF) Testing: A 4-way 2-week model generated in male and female Long Evans rats was grouped as follows: CON: control diet + air exposure; ETOH: ethanol diet + air exposure; CS: control diet + CS exposure; and ETOH + CS: ethanol diet + CS exposure. OF test was performed after 2 weeks of exposure and data were analyzed by EthoVision XT v16 software with respect to (a) the percentage of time spent in the center; (b) the percentage of entries into the center; (c) the latency to the center (sec); (d) the movement distance (cm) in the open field. (e) EthoVision generated heatmaps depict location of each rat in the arena during analysis. The box and whisker plots depict the mean and 95% confidence intervals of the parameters. Inter-group comparisons were made by two-way ANOVA with the Tukey post hoc tests (*P < 0.05; **P < 0.01; ***P < 0.001; δ0.05 < P < 0.10).
